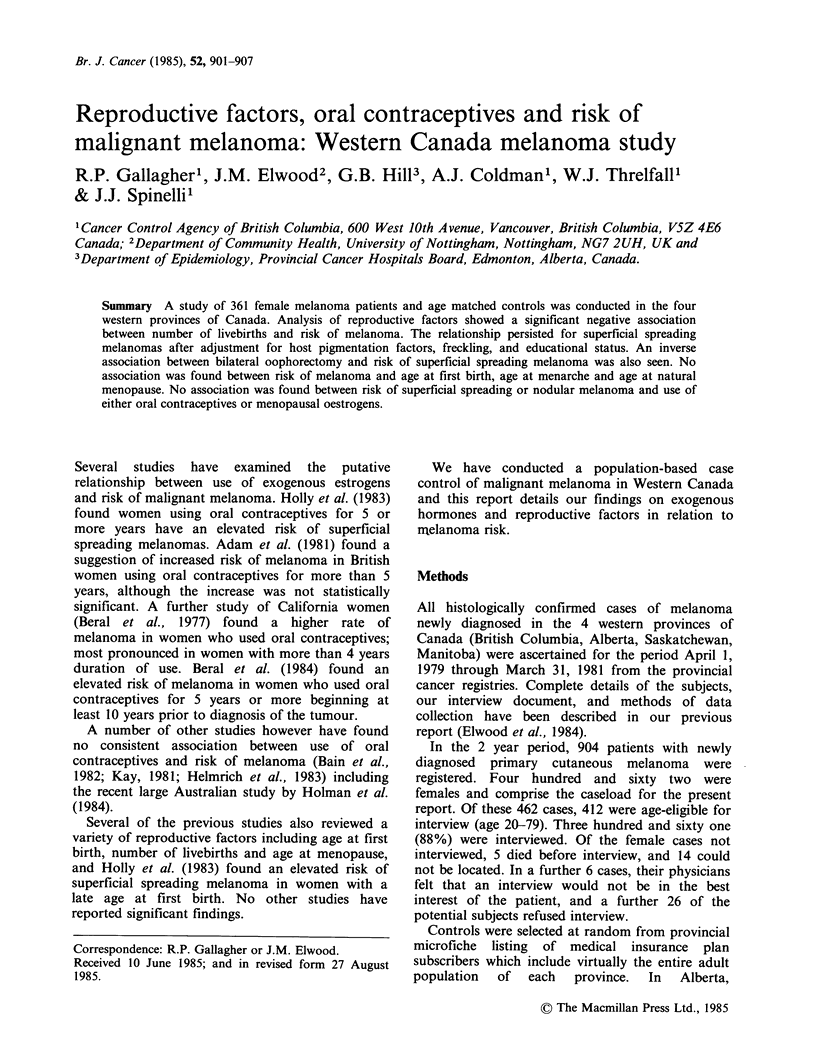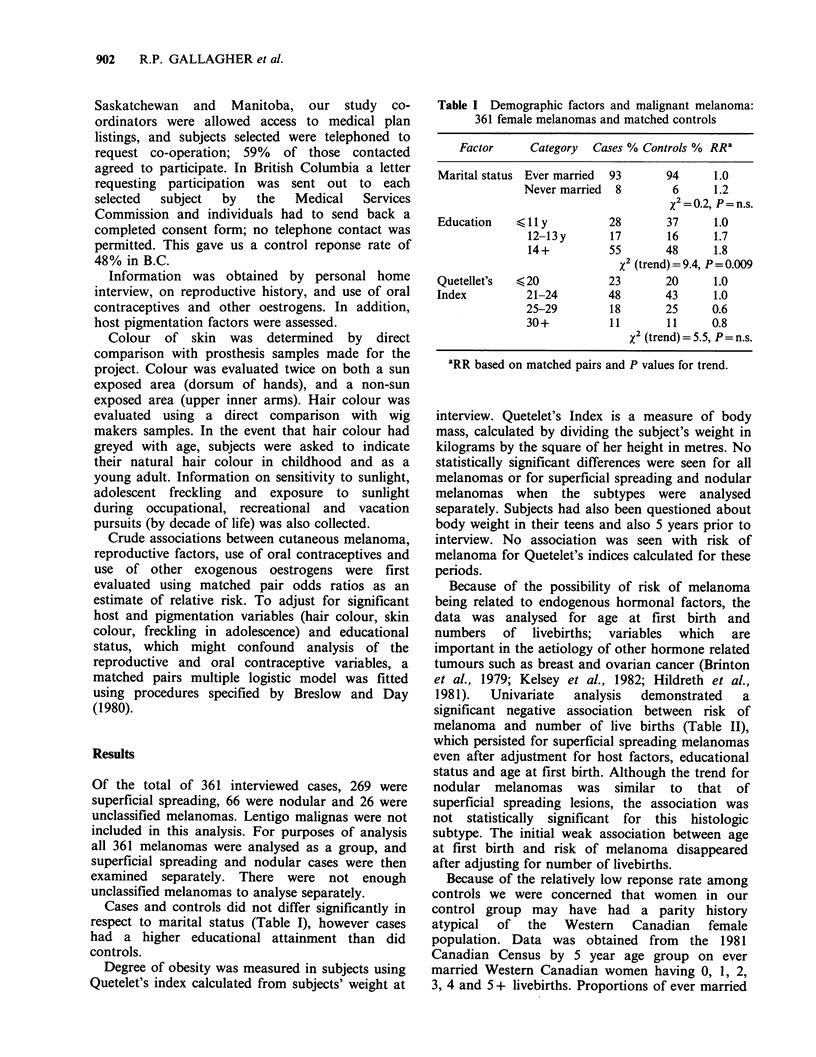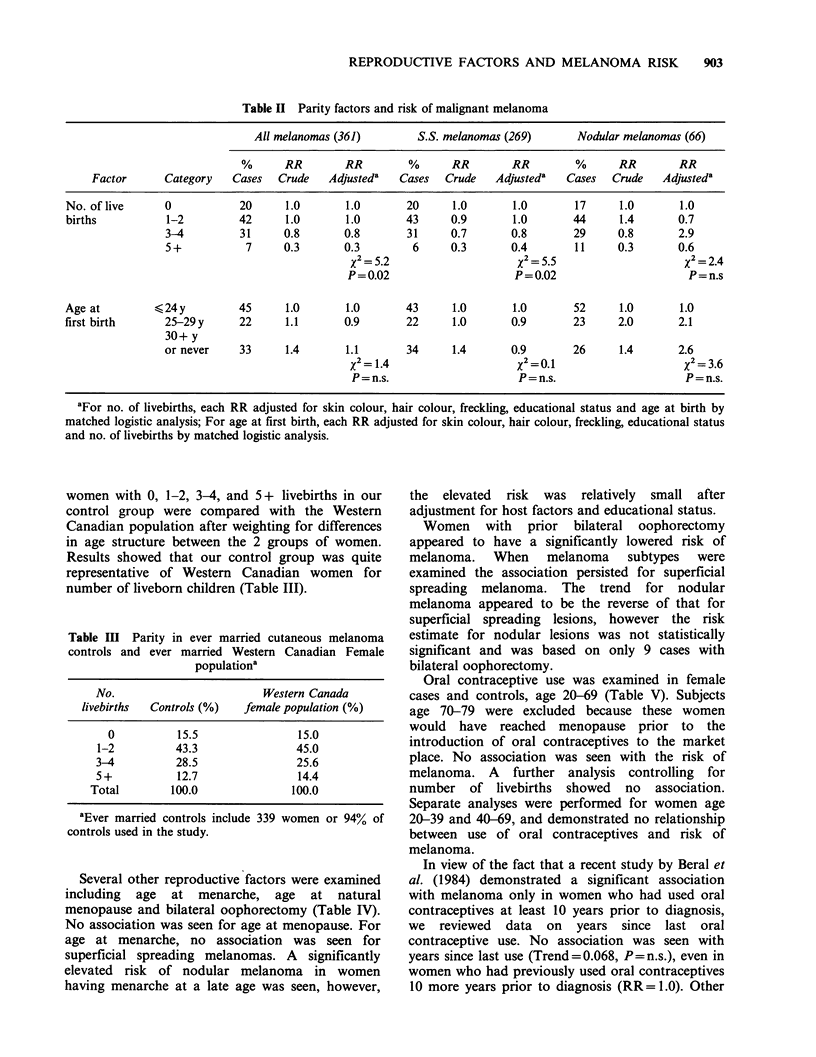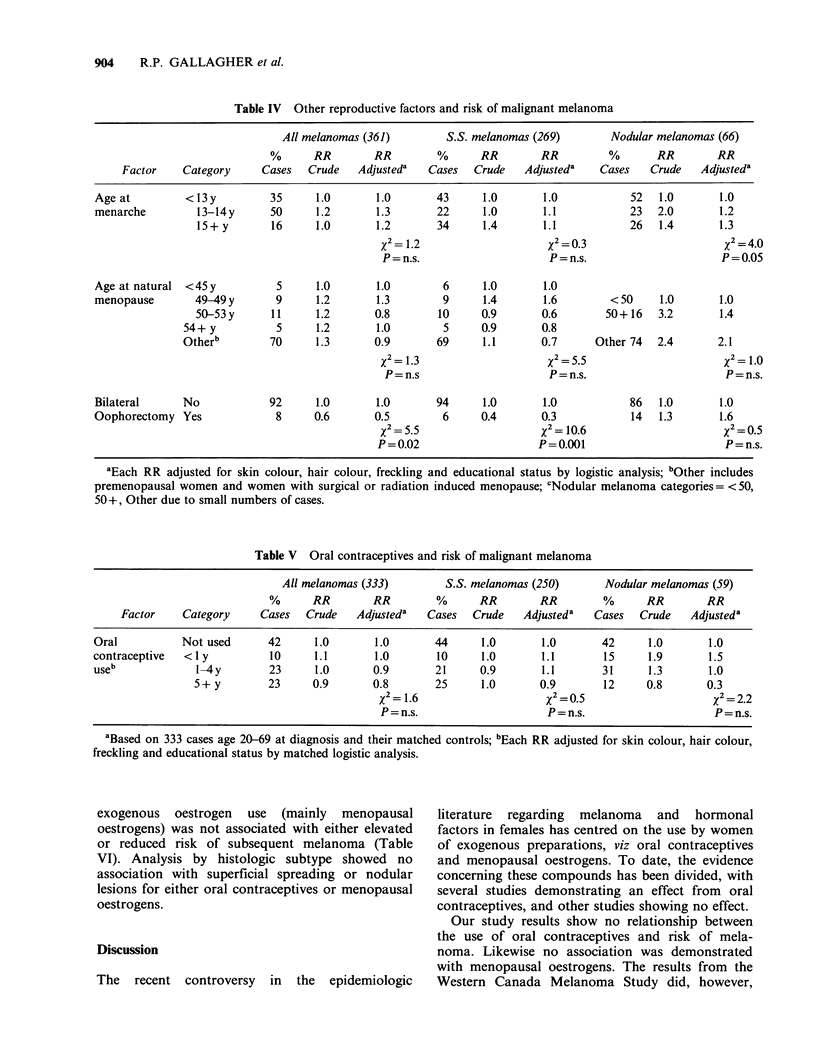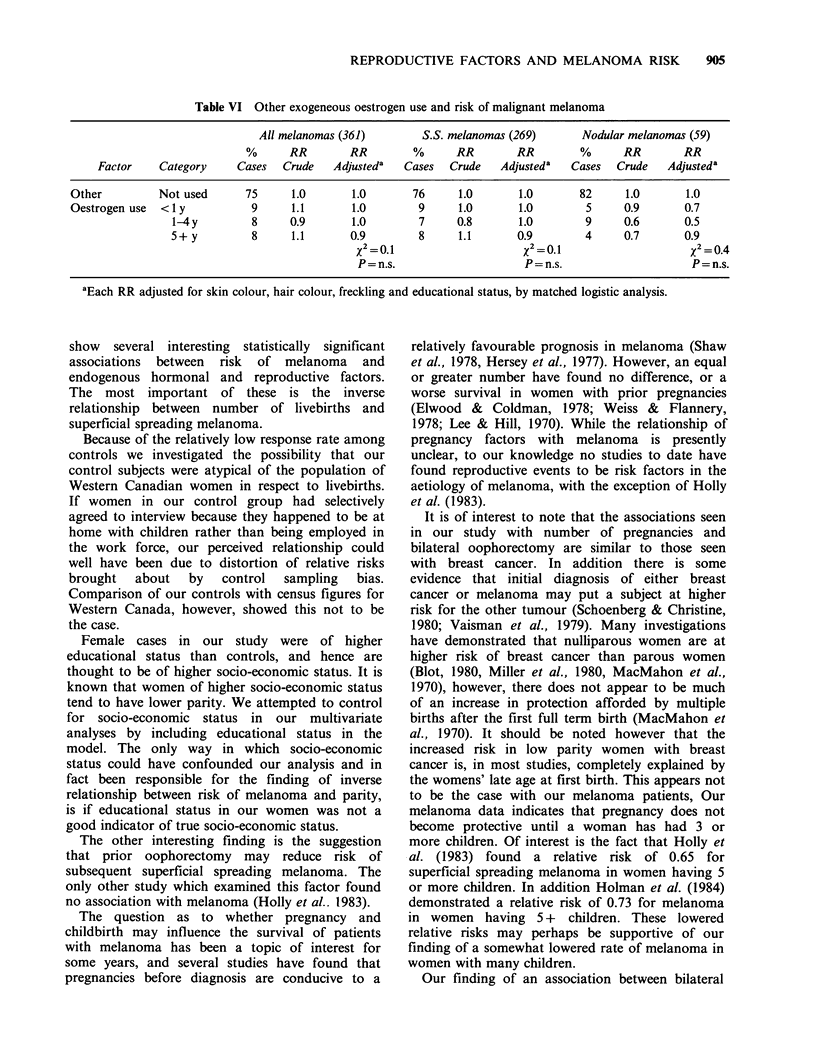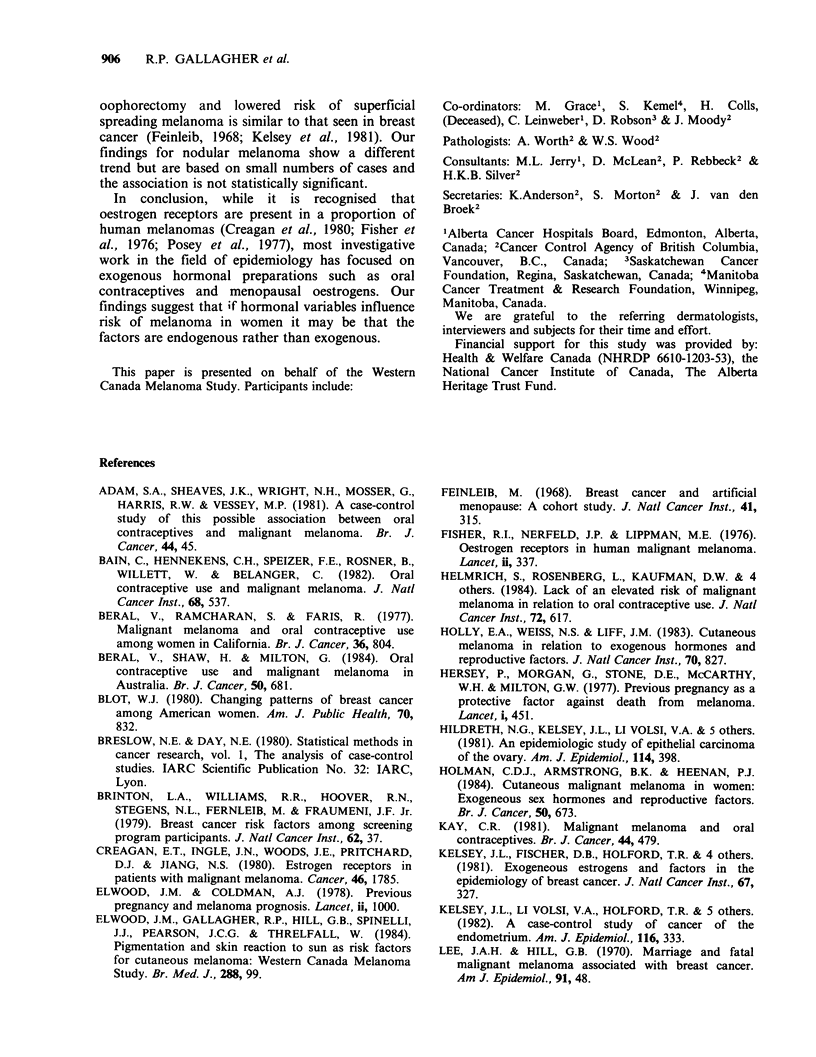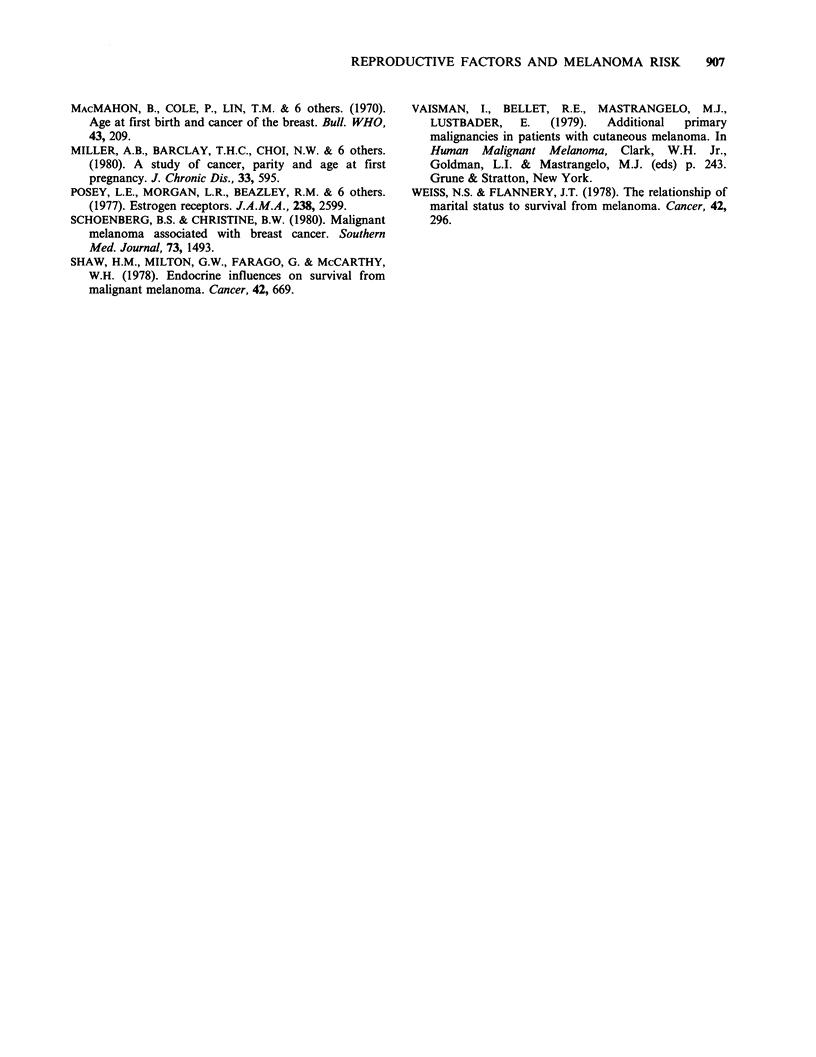# Reproductive factors, oral contraceptives and risk of malignant melanoma: Western Canada Melanoma Study.

**DOI:** 10.1038/bjc.1985.275

**Published:** 1985-12

**Authors:** R. P. Gallagher, J. M. Elwood, G. B. Hill, A. J. Coldman, W. J. Threlfall, J. J. Spinelli

## Abstract

A study of 361 female melanoma patients and age matched controls was conducted in the four western provinces of Canada. Analysis of reproductive factors showed a significant negative association between number of livebirths and risk of melanoma. The relationship persisted for superficial spreading melanomas after adjustment for host pigmentation factors, freckling, and educational status. An inverse association between bilateral oophorectomy and risk of superficial spreading melanoma was also seen. No association was found between risk of melanoma and age at first birth, age at menarche and age at natural menopause. No association was found between risk of superficial spreading or nodular melanoma and use of either oral contraceptives or menopausal oestrogens.


*Br. J. Cancer* (1985), **52**, 901–907

### Reproductive factors, oral contraceptives and risk of malignant melanoma: Western Canada melanoma study

R.P. Gallagher[1], J.M. Elwood[2], G.B. Hill[3], A.J. Coldman[1], W.J. Threlfall[1] & J.J. Spinelli[1]

[1]*Cancer Control Agency of British Columbia, 600 West 10th Avenue, Vancouver, British Columbia, V5Z 4E6 Canada;* [2]*Department of Community Health, University of Nottingham, Nottingham, NG7 2UH, UK and* [3]*Department of Epidemiology, Provincial Cancer Hospitals Board, Edmonton, Alberta, Canada.*

**Summary** A study of 361 female melanoma patients and age matched controls was conducted in the four western provinces of Canada. Analysis of reproductive factors showed a significant negative association between number of livebirths and risk of melanoma. The relationship persisted for superficial spreading melanomas after adjustment for host pigmentation factors, freckling, and educational status. An inverse association between bilateral oophorectomy and risk of superficial spreading melanoma was also seen. No association was found between risk of melanoma and age at first birth, age at menarche and age at natural menopause. No association was found between risk of superficial spreading or nodular melanoma and use of either oral contraceptives or menopausal oestrogens.

Several studies have examined the putative relationship between use of exogenous estrogens and risk of malignant melanoma. Holly *et al.* (1983) found women using oral contraceptives for 5 or more years have an elevated risk of superficial spreading melanomas. Adam *et al.* (1981) found a suggestion of increased risk of melanoma in British women using oral contraceptives for more than 5 years, although the increase was not statistically significant. A further study of California women (Beral *et al.*, 1977) found a higher rate of melanoma in women who used oral contraceptives; most pronounced in women with more than 4 years duration of use. Beral *et al.* (1984) found an elevated risk of melanoma in women who used oral contraceptives for 5 years or more beginning at least 10 years prior to diagnosis of the tumour.

A number of other studies however have found no consistent association between use of oral contraceptives and risk of melanoma (Bain *et al.*, 1982; Kay, 1981; Helmrich *et al.*, 1983) including the recent large Australian study by Holman *et al.* (1984).

Several of the previous studies also reviewed a variety of reproductive factors including age at first birth, number of livebirths and age at menopause, and Holly *et al.* (1983) found an elevated risk of superficial spreading melanoma in women with a late age at first birth. No other studies have reported significant findings.

Correspondence: R.P. Gallagher or J.M. Elwood.
Received 10 June 1985; and in revised form 27 August 1985.

We have conducted a population-based case control of malignant melanoma in Western Canada and this report details our findings on exogenous hormones and reproductive factors in relation to melanoma risk.

### Methods

All histologically confirmed cases of melanoma newly diagnosed in the 4 western provinces of Canada (British Columbia, Alberta, Saskatchewan, Manitoba) were ascertained for the period April 1, 1979 through March 31, 1981 from the provincial cancer registries. Complete details of the subjects, our interview document, and methods of data collection have been described in our previous report (Elwood *et al.*, 1984).

In the 2 year period, 904 patients with newly diagnosed primary cutaneous melanoma were registered. Four hundred and sixty two were females and comprise the caseload for the present report. Of these 462 cases, 412 were age-eligible for interview (age 20–79). Three hundred and sixty one (88%) were interviewed. Of the female cases not interviewed, 5 died before interview, and 14 could not be located. In a further 6 cases, their physicians felt that an interview would not be in the best interest of the patient, and a further 26 of the potential subjects refused interview.

Controls were selected at random from provincial microfiche listing of medical insurance plan subscribers which include virtually the entire adult population of each province. In Alberta,

© The Macmillan Press Ltd., 1985

Saskatchewan and Manitoba, our study co-ordinators were allowed access to medical plan listings, and subjects selected were telephoned to request co-operation; 59% of those contacted agreed to participate. In British Columbia a letter requesting participation was sent out to each selected subject by the Medical Services Commission and individuals had to send back a completed consent form; no telephone contact was permitted. This gave us a control reponse rate of 48% in B.C.

Information was obtained by personal home interview, on reproductive history, and use of oral contraceptives and other oestrogens. In addition, host pigmentation factors were assessed.

Colour of skin was determined by direct comparison with prosthesis samples made for the project. Colour was evaluated twice on both a sun exposed area (dorsum of hands), and a non-sun exposed area (upper inner arms). Hair colour was evaluated using a direct comparison with wig makers samples. In the event that hair colour had greyed with age, subjects were asked to indicate their natural hair colour in childhood and as a young adult. Information on sensitivity to sunlight, adolescent freckling and exposure to sunlight during occupational, recreational and vacation pursuits (by decade of life) was also collected.

Crude associations between cutaneous melanoma, reproductive factors, use of oral contraceptives and use of other exogenous oestrogens were first evaluated using matched pair odds ratios as an estimate of relative risk. To adjust for significant host and pigmentation variables (hair colour, skin colour, freckling in adolescence) and educational status, which might confound analysis of the reproductive and oral contraceptive variables, a matched pairs multiple logistic model was fitted using procedures specified by Breslow and Day (1980).

**Table I** Demographic factors and malignant melanoma: 361 female melanomas and matched controls

| *Factor* | *Category* | *Cases %* | *Controls %* | *RR*[a] |
|---|---|---|---|---|
| Marital status | Ever married | 93 | 94 | 1.0 |
| | Never married | 8 | 6 | 1.2 |
| | | | $\chi^2=0.2$, $P=$n.s. | |
| Education | ⩽11 y | 28 | 37 | 1.0 |
| | 12–13 y | 17 | 16 | 1.7 |
| | 14+ | 55 | 48 | 1.8 |
| | | | $\chi^2$ (trend) $=9.4$, $P=0.009$ | |
| Quetellet's Index | ⩽20 | 23 | 20 | 1.0 |
| | 21–24 | 48 | 43 | 1.0 |
| | 25–29 | 18 | 25 | 0.6 |
| | 30+ | 11 | 11 | 0.8 |
| | | | $\chi^2$ (trend) $=5.5$, $P=$n.s. | |

[a]RR based on matched pairs and *P* values for trend.

### Results

Of the total of 361 interviewed cases, 269 were superficial spreading, 66 were nodular and 26 were unclassified melanomas. Lentigo malignas were not included in this analysis. For purposes of analysis all 361 melanomas were analysed as a group, and superficial spreading and nodular cases were then examined separately. There were not enough unclassified melanomas to analyse separately.

Cases and controls did not differ significantly in respect to marital status (Table I), however cases had a higher educational attainment than did controls.

Degree of obesity was measured in subjects using Quetelet's index calculated from subjects' weight at interview. Quetelet's Index is a measure of body mass, calculated by dividing the subject's weight in kilograms by the square of her height in metres. No statistically significant differences were seen for all melanomas or for superficial spreading and nodular melanomas when the subtypes were analysed separately. Subjects had also been questioned about body weight in their teens and also 5 years prior to interview. No association was seen with risk of melanoma for Quetelet's indices calculated for these periods.

Because of the possibility of risk of melanoma being related to endogenous hormonal factors, the data was analysed for age at first birth and numbers of livebirths; variables which are important in the aetiology of other hormone related tumours such as breast and ovarian cancer (Brinton *et al.*, 1979; Kelsey *et al.*, 1982; Hildreth *et al.*, 1981). Univariate analysis demonstrated a significant negative association between risk of melanoma and number of live births (Table II), which persisted for superficial spreading melanomas even after adjustment for host factors, educational status and age at first birth. Although the trend for nodular melanomas was similar to that of superficial spreading lesions, the association was not statistically significant for this histologic subtype. The initial weak association between age at first birth and risk of melanoma disappeared after adjusting for number of livebirths.

Because of the relatively low reponse rate among controls we were concerned that women in our control group may have had a parity history atypical of the Western Canadian female population. Data was obtained from the 1981 Canadian Census by 5 year age group on ever married Western Canadian women having 0, 1, 2, 3, 4 and 5+ livebirths. Proportions of ever married

**Table II** Parity factors and risk of malignant melanoma

| | | *All melanomas (361)* | | | *S.S. melanomas (269)* | | | *Nodular melanomas (66)* | | |
|---|---|---|---|---|---|---|---|---|---|---|
| *Factor* | *Category* | *% Cases* | *RR Crude* | *RR Adjusted*[a] | *% Cases* | *RR Crude* | *RR Adjusted*[a] | *% Cases* | *RR Crude* | *RR Adjusted*[a] |
| No. of live births | 0 | 20 | 1.0 | 1.0 | 20 | 1.0 | 1.0 | 17 | 1.0 | 1.0 |
| | 1–2 | 42 | 1.0 | 1.0 | 43 | 0.9 | 1.0 | 44 | 1.4 | 0.7 |
| | 3–4 | 31 | 0.8 | 0.8 | 31 | 0.7 | 0.8 | 29 | 0.8 | 2.9 |
| | 5+ | 7 | 0.3 | 0.3 | 6 | 0.3 | 0.4 | 11 | 0.3 | 0.6 |
| | | | | $\chi^2=5.2$ | | | $\chi^2=5.5$ | | | $\chi^2=2.4$ |
| | | | | $P=0.02$ | | | $P=0.02$ | | | $P=$n.s |
| Age at first birth | ≤24 y | 45 | 1.0 | 1.0 | 43 | 1.0 | 1.0 | 52 | 1.0 | 1.0 |
| | 25–29 y | 22 | 1.1 | 0.9 | 22 | 1.0 | 0.9 | 23 | 2.0 | 2.1 |
| | 30+ y or never | 33 | 1.4 | 1.1 | 34 | 1.4 | 0.9 | 26 | 1.4 | 2.6 |
| | | | | $\chi^2=1.4$ | | | $\chi^2=0.1$ | | | $\chi^2=3.6$ |
| | | | | $P=$n.s. | | | $P=$n.s. | | | $P=$n.s. |

[a]For no. of livebirths, each RR adjusted for skin colour, hair colour, freckling, educational status and age at birth by matched logistic analysis; For age at first birth, each RR adjusted for skin colour, hair colour, freckling, educational status and no. of livebirths by matched logistic analysis.

women with 0, 1–2, 3–4, and 5+ livebirths in our control group were compared with the Western Canadian population after weighting for differences in age structure between the 2 groups of women. Results showed that our control group was quite representative of Western Canadian women for number of liveborn children (Table III).

**Table III** Parity in ever married cutaneous melanoma controls and ever married Western Canadian Female population[a]

| *No. livebirths* | *Controls* (%) | *Western Canada female population* (%) |
|---|---|---|
| 0 | 15.5 | 15.0 |
| 1–2 | 43.3 | 45.0 |
| 3–4 | 28.5 | 25.6 |
| 5+ | 12.7 | 14.4 |
| Total | 100.0 | 100.0 |

[a]Ever married controls include 339 women or 94% of controls used in the study.

Several other reproductive factors were examined including age at menarche, age at natural menopause and bilateral oophorectomy (Table IV). No association was seen for age at menopause. For age at menarche, no association was seen for superficial spreading melanomas. A significantly elevated risk of nodular melanoma in women having menarche at a late age was seen, however, the elevated risk was relatively small after adjustment for host factors and educational status.

Women with prior bilateral oophorectomy appeared to have a significantly lowered risk of melanoma. When melanoma subtypes were examined the association persisted for superficial spreading melanoma. The trend for nodular melanoma appeared to be the reverse of that for superficial spreading lesions, however the risk estimate for nodular lesions was not statistically significant and was based on only 9 cases with bilateral oophorectomy.

Oral contraceptive use was examined in female cases and controls, age 20–69 (Table V). Subjects age 70–79 were excluded because these women would have reached menopause prior to the introduction of oral contraceptives to the market place. No association was seen with the risk of melanoma. A further analysis controlling for number of livebirths showed no association. Separate analyses were performed for women age 20–39 and 40–69, and demonstrated no relationship between use of oral contraceptives and risk of melanoma.

In view of the fact that a recent study by Beral *et al.* (1984) demonstrated a significant association with melanoma only in women who had used oral contraceptives at least 10 years prior to diagnosis, we reviewed data on years since last oral contraceptive use. No association was seen with years since last use (Trend = 0.068, $P=$n.s.), even in women who had previously used oral contraceptives 10 more years prior to diagnosis (RR = 1.0). Other

**Table IV** Other reproductive factors and risk of malignant melanoma

| | | *All melanomas (361)* | | | *S.S. melanomas (269)* | | | *Nodular melanomas (66)* | | |
|---|---|---|---|---|---|---|---|---|---|---|
| *Factor* | *Category* | *% Cases* | *RR Crude* | *RR Adjusted*[a] | *% Cases* | *RR Crude* | *RR Adjusted*[a] | *% Cases* | *RR Crude* | *RR Adjusted*[a] |
| Age at menarche | <13 y | 35 | 1.0 | 1.0 | 43 | 1.0 | 1.0 | 52 | 1.0 | 1.0 |
| | 13–14 y | 50 | 1.2 | 1.3 | 22 | 1.0 | 1.1 | 23 | 2.0 | 1.2 |
| | 15+ y | 16 | 1.0 | 1.2 | 34 | 1.4 | 1.1 | 26 | 1.4 | 1.3 |
| | | | | $\chi^2=1.2$ | | | $\chi^2=0.3$ | | | $\chi^2=4.0$ |
| | | | | $P$=n.s. | | | $P$=n.s. | | | $P=0.05$ |
| Age at natural menopause | <45 y | 5 | 1.0 | 1.0 | 6 | 1.0 | 1.0 | | | |
| | 49–49 y | 9 | 1.2 | 1.3 | 9 | 1.4 | 1.6 | <50 | 1.0 | 1.0 |
| | 50–53 y | 11 | 1.2 | 0.8 | 10 | 0.9 | 0.6 | 50+ 16 | 3.2 | 1.4 |
| | 54+ y | 5 | 1.2 | 1.0 | 5 | 0.9 | 0.8 | | | |
| | Other[b] | 70 | 1.3 | 0.9 | 69 | 1.1 | 0.7 | Other 74 | 2.4 | 2.1 |
| | | | | $\chi^2=1.3$ | | | $\chi^2=5.5$ | | | $\chi^2=1.0$ |
| | | | | $P$=n.s | | | $P$=n.s. | | | $P$=n.s. |
| Bilateral Oophorectomy | No | 92 | 1.0 | 1.0 | 94 | 1.0 | 1.0 | 86 | 1.0 | 1.0 |
| | Yes | 8 | 0.6 | 0.5 | 6 | 0.4 | 0.3 | 14 | 1.3 | 1.6 |
| | | | | $\chi^2=5.5$ | | | $\chi^2=10.6$ | | | $\chi^2=0.5$ |
| | | | | $P=0.02$ | | | $P=0.001$ | | | $P$=n.s. |

[a]Each RR adjusted for skin colour, hair colour, freckling and educational status by logistic analysis; [b]Other includes premenopausal women and women with surgical or radiation induced menopause; [c]Nodular melanoma categories = <50, 50+, Other due to small numbers of cases.

**Table V** Oral contraceptives and risk of malignant melanoma

| | | *All melanomas (333)* | | | *S.S. melanomas (250)* | | | *Nodular melanomas (59)* | | |
|---|---|---|---|---|---|---|---|---|---|---|
| *Factor* | *Category* | *% Cases* | *RR Crude* | *RR Adjusted*[a] | *% Cases* | *RR Crude* | *RR Adjusted*[a] | *% Cases* | *RR Crude* | *RR Adjusted*[a] |
| Oral contraceptive use[b] | Not used | 42 | 1.0 | 1.0 | 44 | 1.0 | 1.0 | 42 | 1.0 | 1.0 |
| | <1 y | 10 | 1.1 | 1.0 | 10 | 1.0 | 1.1 | 15 | 1.9 | 1.5 |
| | 1–4 y | 23 | 1.0 | 0.9 | 21 | 0.9 | 1.1 | 31 | 1.3 | 1.0 |
| | 5+ y | 23 | 0.9 | 0.8 | 25 | 1.0 | 0.9 | 12 | 0.8 | 0.3 |
| | | | | $\chi^2=1.6$ | | | $\chi^2=0.5$ | | | $\chi^2=2.2$ |
| | | | | $P$=n.s. | | | $P$=n.s. | | | $P$=n.s. |

[a]Based on 333 cases age 20–69 at diagnosis and their matched controls; [b]Each RR adjusted for skin colour, hair colour, freckling and educational status by matched logistic analysis.

exogenous oestrogen use (mainly menopausal oestrogens) was not associated with either elevated or reduced risk of subsequent melanoma (Table VI). Analysis by histologic subtype showed no association with superficial spreading or nodular lesions for either oral contraceptives or menopausal oestrogens.

### Discussion

The recent controversy in the epidemiologic literature regarding melanoma and hormonal factors in females has centred on the use by women of exogenous preparations, *viz* oral contraceptives and menopausal oestrogens. To date, the evidence concerning these compounds has been divided, with several studies demonstrating an effect from oral contraceptives, and other studies showing no effect.

Our study results show no relationship between the use of oral contraceptives and risk of melanoma. Likewise no association was demonstrated with menopausal oestrogens. The results from the Western Canada Melanoma Study did, however,

**Table VI** Other exogeneous oestrogen use and risk of malignant melanoma

| Factor | Category | *All melanomas (361)* % Cases | RR Crude | RR Adjusted[a] | *S.S. melanomas (269)* % Cases | RR Crude | RR Adjusted[a] | *Nodular melanomas (59)* % Cases | RR Crude | RR Adjusted[a] |
|---|---|---|---|---|---|---|---|---|---|---|
| Other | Not used | 75 | 1.0 | 1.0 | 76 | 1.0 | 1.0 | 82 | 1.0 | 1.0 |
| Oestrogen use | <1 y | 9 | 1.1 | 1.0 | 9 | 1.0 | 1.0 | 5 | 0.9 | 0.7 |
| | 1–4 y | 8 | 0.9 | 1.0 | 7 | 0.8 | 1.0 | 9 | 0.6 | 0.5 |
| | 5+ y | 8 | 1.1 | 0.9 | 8 | 1.1 | 0.9 | 4 | 0.7 | 0.9 |
| | | | | $\chi^2=0.1$ | | | $\chi^2=0.1$ | | | $\chi^2=0.4$ |
| | | | | $P$ = n.s. | | | $P$ = n.s. | | | $P$ = n.s. |

[a]Each RR adjusted for skin colour, hair colour, freckling and educational status, by matched logistic analysis.

show several interesting statistically significant associations between risk of melanoma and endogenous hormonal and reproductive factors. The most important of these is the inverse relationship between number of livebirths and superficial spreading melanoma.

Because of the relatively low response rate among controls we investigated the possibility that our control subjects were atypical of the population of Western Canadian women in respect to livebirths. If women in our control group had selectively agreed to interview because they happened to be at home with children rather than being employed in the work force, our perceived relationship could well have been due to distortion of relative risks brought about by control sampling bias. Comparison of our controls with census figures for Western Canada, however, showed this not to be the case.

Female cases in our study were of higher educational status than controls, and hence are thought to be of higher socio-economic status. It is known that women of higher socio-economic status tend to have lower parity. We attempted to control for socio-economic status in our multivariate analyses by including educational status in the model. The only way in which socio-economic status could have confounded our analysis and in fact been responsible for the finding of inverse relationship between risk of melanoma and parity, is if educational status in our women was not a good indicator of true socio-economic status.

The other interesting finding is the suggestion that prior oophorectomy may reduce risk of subsequent superficial spreading melanoma. The only other study which examined this factor found no association with melanoma (Holly *et al*.. 1983).

The question as to whether pregnancy and childbirth may influence the survival of patients with melanoma has been a topic of interest for some years, and several studies have found that pregnancies before diagnosis are conducive to a relatively favourable prognosis in melanoma (Shaw *et al.*, 1978, Hersey *et al.*, 1977). However, an equal or greater number have found no difference, or a worse survival in women with prior pregnancies (Elwood & Coldman, 1978; Weiss & Flannery, 1978; Lee & Hill, 1970). While the relationship of pregnancy factors with melanoma is presently unclear, to our knowledge no studies to date have found reproductive events to be risk factors in the aetiology of melanoma, with the exception of Holly *et al.* (1983).

It is of interest to note that the associations seen in our study with number of pregnancies and bilateral oophorectomy are similar to those seen with breast cancer. In addition there is some evidence that initial diagnosis of either breast cancer or melanoma may put a subject at higher risk for the other tumour (Schoenberg & Christine, 1980; Vaisman *et al.*, 1979). Many investigations have demonstrated that nulliparous women are at higher risk of breast cancer than parous women (Blot, 1980, Miller *et al.*, 1980, MacMahon *et al.*, 1970), however, there does not appear to be much of an increase in protection afforded by multiple births after the first full term birth (MacMahon *et al.*, 1970). It should be noted however that the increased risk in low parity women with breast cancer is, in most studies, completely explained by the womens' late age at first birth. This appears not to be the case with our melanoma patients, Our melanoma data indicates that pregnancy does not become protective until a woman has had 3 or more children. Of interest is the fact that Holly *et al.* (1983) found a relative risk of 0.65 for superficial spreading melanoma in women having 5 or more children. In addition Holman *et al.* (1984) demonstrated a relative risk of 0.73 for melanoma in women having 5+ children. These lowered relative risks may perhaps be supportive of our finding of a somewhat lowered rate of melanoma in women with many children.

Our finding of an association between bilateral oophorectomy and lowered risk of superficial spreading melanoma is similar to that seen in breast cancer (Feinleib, 1968; Kelsey *et al.*, 1981). Our findings for nodular melanoma show a different trend but are based on small numbers of cases and the association is not statistically significant.

In conclusion, while it is recognised that oestrogen receptors are present in a proportion of human melanomas (Creagan *et al.*, 1980; Fisher *et al.*, 1976; Posey *et al.*, 1977), most investigative work in the field of epidemiology has focused on exogenous hormonal preparations such as oral contraceptives and menopausal oestrogens. Our findings suggest that if hormonal variables influence risk of melanoma in women it may be that the factors are endogenous rather than exogenous.

This paper is presented on behalf of the Western Canada Melanoma Study. Participants include:

Co-ordinators: M. Grace[1], S. Kemel[4], H. Colls, (Deceased), C. Leinweber[1], D. Robson[3] & J. Moody[2]

Pathologists: A. Worth[2] & W.S. Wood[2]

Consultants: M.L. Jerry[1], D. McLean[2], P. Rebbeck[2] & H.K.B. Silver[2]

Secretaries: K.Anderson[2], S. Morton[2] & J. van den Broek[2]

[1]Alberta Cancer Hospitals Board, Edmonton, Alberta, Canada; [2]Cancer Control Agency of British Columbia, Vancouver, B.C., Canada; [3]Saskatchewan Cancer Foundation, Regina, Saskatchewan, Canada; [4]Manitoba Cancer Treatment & Research Foundation, Winnipeg, Manitoba, Canada.

We are grateful to the referring dermatologists, interviewers and subjects for their time and effort.

Financial support for this study was provided by: Health & Welfare Canada (NHRDP 6610-1203-53), the National Cancer Institute of Canada, The Alberta Heritage Trust Fund.

### References

ADAM, S.A., SHEAVES, J.K., WRIGHT, N.H., MOSSER, G., HARRIS, R.W. & VESSEY, M.P. (1981). A case-control study of this possible association between oral contraceptives and malignant melanoma. *Br. J. Cancer*, **44**, 45.

BAIN, C., HENNEKENS, C.H., SPEIZER, F.E., ROSNER, B., WILLETT, W. & BELANGER, C. (1982). Oral contraceptive use and malignant melanoma. *J. Natl Cancer Inst.*, **68**, 537.

BERAL, V., RAMCHARAN, S. & FARIS, R. (1977). Malignant melanoma and oral contraceptive use among women in California. *Br. J. Cancer*, **36**, 804.

BERAL, V., SHAW, H. & MILTON, G. (1984). Oral contraceptive use and malignant melanoma in Australia. *Br. J. Cancer*, **50**, 681.

BLOT, W.J. (1980). Changing patterns of breast cancer among American women. *Am. J. Public Health*, **70**, 832.

BRESLOW, N.E. & DAY, N.E. (1980). Statistical methods in cancer research, vol. 1, The analysis of case-control studies. IARC Scientific Publication No. 32: IARC, Lyon.

BRINTON, L.A., WILLIAMS, R.R., HOOVER, R.N., STEGENS, N.L., FERNLEIB, M. & FRAUMENI, J.F. Jr. (1979). Breast cancer risk factors among screening program participants. *J. Natl Cancer Inst.*, **62**, 37.

CREAGAN, E.T., INGLE, J.N., WOODS, J.E., PRITCHARD, D.J. & JIANG, N.S. (1980). Estrogen receptors in patients with malignant melanoma. *Cancer*, **46**, 1785.

ELWOOD, J.M. & COLDMAN, A.J. (1978). Previous pregnancy and melanoma prognosis. *Lancet*, **ii**, 1000.

ELWOOD, J.M., GALLAGHER, R.P., HILL, G.B., SPINELLI, J.J., PEARSON, J.C.G. & THRELFALL, W. (1984). Pigmentation and skin reaction to sun as risk factors for cutaneous melanoma: Western Canada Melanoma Study. *Br. Med. J.*, **288**, 99.

FEINLEIB, M. (1968). Breast cancer and artificial menopause: A cohort study. *J. Natl Cancer Inst.*, **41**, 315.

FISHER, R.I., NERFELD, J.P. & LIPPMAN, M.E. (1976). Oestrogen receptors in human malignant melanoma. *Lancet*, **ii**, 337.

HELMRICH, S., ROSENBERG, L., KAUFMAN, D.W. & 4 others. (1984). Lack of an elevated risk of malignant melanoma in relation to oral contraceptive use. *J. Natl Cancer Inst.*, **72**, 617.

HOLLY, E.A., WEISS, N.S. & LIFF, J.M. (1983). Cutaneous melanoma in relation to exogenous hormones and reproductive factors. *J. Natl Cancer Inst.*, **70**, 827.

HERSEY, P., MORGAN, G., STONE, D.E., McCARTHY, W.H. & MILTON, G.W. (1977). Previous pregnancy as a protective factor against death from melanoma. *Lancet*, **i**, 451.

HILDRETH, N.G., KELSEY, J.L., LI VOLSI, V.A. & 5 others. (1981). An epidemiologic study of epithelial carcinoma of the ovary. *Am. J. Epidemiol.*, **114**, 398.

HOLMAN, C.D.J., ARMSTRONG, B.K. & HEENAN, P.J. (1984). Cutaneous malignant melanoma in women: Exogeneous sex hormones and reproductive factors. *Br. J. Cancer*, **50**, 673.

KAY, C.R. (1981). Malignant melanoma and oral contraceptives. *Br. J. Cancer*, **44**, 479.

KELSEY, J.L., FISCHER, D.B., HOLFORD, T.R. & 4 others. (1981). Exogeneous estrogens and factors in the epidemiology of breast cancer. *J. Natl Cancer Inst.*, **67**, 327.

KELSEY, J.L., LI VOLSI, V.A., HOLFORD, T.R. & 5 others. (1982). A case-control study of cancer of the endometrium. *Am. J. Epidemiol.*, **116**, 333.

LEE, J.A.H. & HILL, G.B. (1970). Marriage and fatal malignant melanoma associated with breast cancer. *Am J. Epidemiol.*, **91**, 48.

MacMAHON, B., COLE, P., LIN, T.M. & 6 others. (1970). Age at first birth and cancer of the breast. *Bull. WHO*, **43**, 209.

MILLER, A.B., BARCLAY, T.H.C., CHOI, N.W. & 6 others. (1980). A study of cancer, parity and age at first pregnancy. *J. Chronic Dis.*, **33**, 595.

POSEY, L.E., MORGAN, L.R., BEAZLEY, R.M. & 6 others. (1977). Estrogen receptors. *J.A.M.A.*, **238**, 2599.

SCHOENBERG, B.S. & CHRISTINE, B.W. (1980). Malignant melanoma associated with breast cancer. *Southern Med. Journal*, **73**, 1493.

SHAW, H.M., MILTON, G.W., FARAGO, G. & McCARTHY, W.H. (1978). Endocrine influences on survival from malignant melanoma. *Cancer*, **42**, 669.

VAISMAN, I., BELLET, R.E., MASTRANGELO, M.J., LUSTBADER, E. (1979). Additional primary malignancies in patients with cutaneous melanoma. In *Human Malignant Melanoma*, Clark, W.H. Jr., Goldman, L.I. & Mastrangelo, M.J. (eds) p. 243. Grune & Stratton, New York.

WEISS, N.S. & FLANNERY, J.T. (1978). The relationship of marital status to survival from melanoma. *Cancer*, **42**, 296.